# A critical role for p38MAPK signalling pathway during reprogramming of human fibroblasts to iPSCs

**DOI:** 10.1038/srep41693

**Published:** 2017-02-03

**Authors:** Irina Neganova, Valeria Chichagova, Lyle Armstrong, Majlinda Lako

**Affiliations:** 1 Institute of Genetic Medicine, Newcastle University, International Centre for Life, NE1 3BZ, UK

## Abstract

Reprogramming of somatic cells to induced pluripotent stem cells (iPSCs) holds enormous promise for regenerative medicine. Reprogramming is a stepwise process with well-defined stages of initiation, maturation and stabilisation which are critically dependent on interactions between key pluripotency transcription factors, epigenetic regulators and signalling pathways. In this manuscript we have investigated the role of p38 MAPK signalling pathway and have shown a subpopulation- and phase-specific pattern of activation occurring during the initiation and maturation stage of reprogramming in partially and fully reprogrammed cells respectively. Downregulation of p38 MAPK activity via RNA interference or small molecule inhibitor led to cell accumulation in G1 phase of the cell cycle and reduced expression of cell cycle regulators during the initiation stage of reprogramming. This was associated with a significant downregulation of key pluripotency marker expression, disruption of mesenchymal to epithelial transition (MET), increased expression of differentiation markers and presence of partially reprogrammed cells which retained a typical gene expression profile of mesendodermal cells and were unable to progress to fully reprogrammed phenotype. Together our data indicate an important role for p38 MAPK activity in proliferation, MET progression and establishment of pluripotent phenotype, which are necessary steps for the development of human iPSCs.

Mitogen-activated protein kinase (MAPK) pathways are activated mainly by environmental stress and cytokine stimuli, generating diverse cellular responses including cell proliferation, differentiation, migration and apoptosis. Four distinct subgroups within MAPKs have been identified including extracellular signal-regulated kinases (ERKs), c-jun N-terminal kinases (JNK/SAPK), ERK/Big MAP kinase 1 (BMK1) and the p38MAPK group of protein kinases. There are four members in the p38 MAPK family: p38α (MAPK14), p38β (MAPK11), p38γ (MAPK12) and p38δ (MAPK13). Activation of the p38 pathway varies in different cells and is dependent on the nature of physiological or stress stimuli. Similarly to other MAPKs, p38 kinases are activated by the mitogen-activated protein kinase kinases (MAPKKs) which include MEKK4, ASK1, ASK2 and TAK1. This in turn causes the activation of map kinases MKK3, MKK6 and to a lesser extent MKK4, which leads to phosphorylation of p38 kinases, targeting substrates in both the cytoplasm and the nucleus. In the cytoplasm, p38 MAPK family members phosphorylate other kinases such as MNK1/2, while in the nucleus they activate a large range of transcription factors (for example ATF2, Elk1, p53 and STAT1) which are involved in DNA damage response, apoptosis, inflammation, developmental processes and cellular proliferation^1^.

Deficiency of p38α in mouse models results in embryonic lethality, due to defective placental organogenesis, suggesting a dispensable role in mouse embryogenesis, whilst being essential for placental development^2^,^3^. Mouse embryonic stem cells (mESCs) lacking p38α and β were generated and shown to be able to differentiate into endothelial, smooth muscle and epithelial cells^4^. Nevertheless their differentiation potential and commitment to cardiomyocytes was compromised^5^.

Contradicting reports exist to date on the role of p38 MAPK during somatic cell reprogramming to generate induced pluripotent stem cells. For example, it has been shown that constant activation of MKK6 is detrimental to the reprogramming of mouse embryonic fibroblasts, whilst activation of MKK3, hyperosomosis driven p38 MAPK activation^6^ or application of a specific p38β inhibitor increases the number of iPSC colonies^7^,^8^, suggesting that the impact of p38 on reprogramming may depend on the mode of its activation. The role of p38 MAPK activity during the reprogramming of human somatic cells has not been studied to date. Furthermore, signalling pathways that maintain and promote pluripotency in human embryonic stem cells (hESCs) and induced pluripotent stem cells (iPSCs) are different to the ones that operate in the mouse system^9^. For example, the MAPK pathway is required to maintain pluripotency and promote self-renewal in hESCs^10^, whereas inhibition of MAPK signalling can support self-renewal of mESCs^11^ which indicates that the role of MAPK signalling during reprogramming of human somatic cells cannot be inferred directly from the mouse cells.

Different components of the p38 pathway participate in tumor suppression by controlling a variety of cellular responses such as replicative senescence, contact inhibition and DNA-damage responses^12^,^13^,^14^,^15^. In normal non-transformed cells, oncogene activation may trigger senescence^16^ which has been shown to provide an effective barrier to iPSC generation^17^. Since Klf4 and c-Myc are known oncogenes, and OCT4 expression has been linked to tumor progression to a cancer stem cell phenotype^18^ it is difficult to exclude involvement of oncogene induced signalling in reprogramming. In accordance with this, it has been shown that constitutively-active HRAS, a member of the Ras oncogene family, significantly reduces iPSC colony generation^7^, whilst inhibition of stress activated JNK/SAPK signalling abrogates human iPSC generation^19^, suggesting that the action of oncogene signalling may be important during various stages of reprogramming. Dissecting the functions of a specific signalling pathway during reprogramming would increase our understanding of the cellular and molecular processes involved in the process and enable identification of new methods to increase its efficiency^19^,^20^. In this manuscript we studied the expression of key components of the p38 MAPK signalling pathway and tested its role in reprogramming by applying small molecule inhibitors or downregulating *p38α* expression using RNA interference. Both approaches point to a critical role for the activity of this pathway during reprogramming of human fibroblasts to iPSCs.

## Results

### Generation of iPSCs by the Yamanaka factors induces activation of p38MAPK

**Figure 1:**
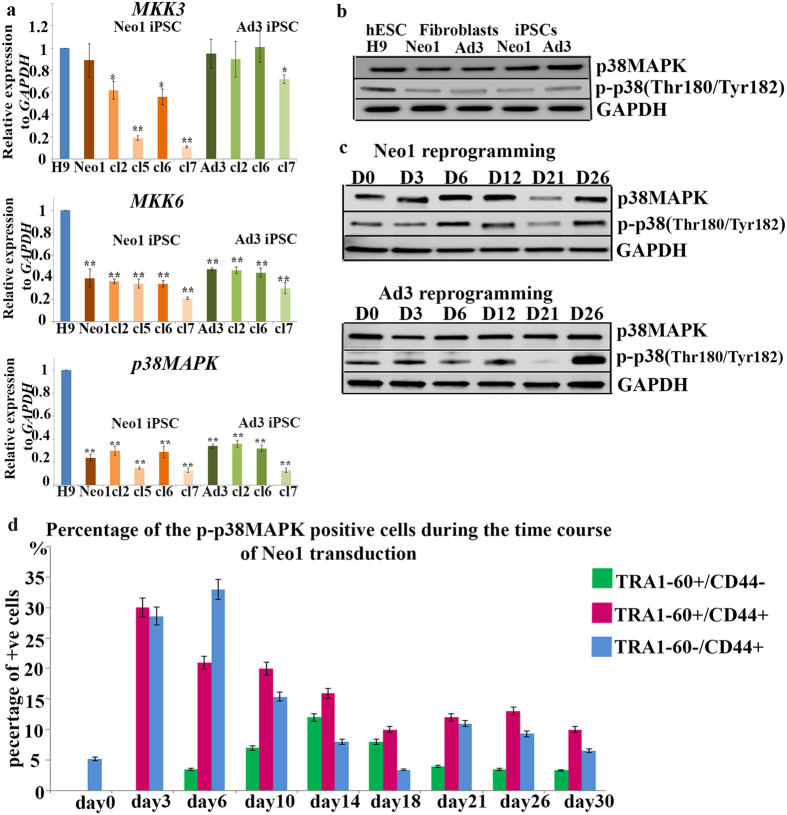
Phase and subpopulation specific p38MAPK signalling during reprogramming of human fibroblasts. (**a**) Quantitave RT-PCR analysis of *MKK3, MKK6* and *p38* MAPK expression in H9 (p35), neonatal human fibroblasts (Neo1), adult human fibroblasts (Ad3) and iPSC generated therefrom (Neo1 clone 2, Neo1 clone 5, Neo1 clone 6, Neo1 clone 7, Ad3 clone 2, Ad3 clone 2 and Ad3 clone 7). Data represent relative expression to *GAPDH* and were normalized against H9. Data are presented as mean ± S.E.M. Student t-test was carried out to detect significant changes between hESCs (H9) and neonatal and adult fibroblasts and respective iPSC. *p < 0.05; **p <  0.01; (**b**) Western blot analysis showing expression of the total form of p38MAPK and phospho p38 (Thr180/Tyr182) MAPK in hESC (H9), neonatal human fibroblasts (Neo1), adult human fibroblasts (Ad3) at Day 0 and iPSC derived therefrom. Cropped images of at least three repeats are shown for purpose of clarity; (**c**) Western blot analysis showing expression of the total form of p38MAPK and phospho-p38 (Thr180/Tyr182) MAPK during the time course of reprogramming of neonatal (Neo1) and adult (Ad3) fibroblasts. Days of transduction = D. GAPDH served as loading control. Images are representative of at least three independent experiments and are cropped; (**d**) Graphic representation of the percentage of p‐p38MAPK positive cells at different cellular populations (TRA1‐60 + /CD44‐, TRA1‐60 + /CD44 + and TRA1‐60‐/CD44 + ) during the reprogramming of neonatal (Neo1) fibroblasts assessed by flow cytometric analysis. Data are presented as mean ± S.E.M, n = 3.

The activation of p38 MAPK signaling by different stress signals is well documented in diverse cellular systems. To clarify if p38 MAPK is activated during the generation of human iPSCs, we analysed the expression of *MKK3, MKK6* and *p38*α expression in neonatal (Neo1) and adult fibroblasts (Ad3), iPSCs clones derived therefrom and hESCs (H9). The expression of *MKK3* did not differ between hESCs, neonatal and adult fibroblasts; however the iPSC clones generated from neonatal fibroblasts demonstrated a lower level of expression (Fig. 1a). Gene expression analysis also revealed reduced levels of *p38a* MAPK in the neonatal and adult fibroblasts, as well as iPSCs clones derived therefrom when compared to hESCs (Fig. 1a). This was also confirmed by western immunoblotting which indicated a reduced expression of a phosphorylated form of p38 MAPK (Thr180/Tyr182) which is indicative of p38MAPK activity^21^ (Fig. 1b). Similarly, a higher level of expression was observed for the upstream activator of p38MAPK, *MKK6,* in hESCs when compared to fibroblasts and iPSC clones (Fig. 1a).

To figure out the involvement of p38 in hiPSC generation, we reprogrammed neonatal and adult fibroblasts with the Yamanaka factors (OSKM) and assessed protein expression of phospho-p38 (Thr180/Tyr182) MAPK during the initiation, maturation and stabilization phases of reprogramming^22^ (Fig. 1c). A biphasic pattern of p38 activation was observed with an initial increase observed at day 6 in neonatal fibroblasts (Neo1) and day 12 for adult fibroblasts (Ad3), followed by a decrease at day 21 and a second increase in expression at day 26 which represents the stabilization stage of reprogramming (Fig. 1c). To identify which subcellular populations expressed the activated form of p38MAPK, we performed flow cytometric analysis at different time points employing the TRA1-60 and CD44 markers to distinguish between fully reprogrammed (TRA1-60^+^CD44^−^) and partially reprogrammed (TRA1-60^+^CD44^+^) cells as described in our recent publication^19^. This analysis indicated a high level of activated p38 MAPK expression at the initiation stages of reprogramming (Day 3) in the partially reprogrammed cells (TRA1-60^+^CD44^+^) which declined during the 30 day time course (Fig. 1d). At the same time the fibroblasts that fail to reprogram (TRA1-60^−^CD44^+^) showed an increase in expression of activated p38MAPK between days 3 and 6 of reprogramming, probably reflecting the cellular response to virus infection. However, this expression declined from day 14 onwards reaching a similar level to that of day 0 by the end of reprogramming period. In contrast, the expression of activated p38 MAPK increased from day 6 to reach peak levels at day 14 of the maturation stage of reprogramming in the fully reprogrammed cells (TRA1-60^+^CD44^−^) before declining gradually towards the end of reprogramming period (Fig. 1d). Together these data indicate a subpopulation- and phase-specific pattern of activated p38 MAPK expression during reprogramming which deserves further in depth investigations as shown in the following results sections.

### Inhibition of p38 MAPK by small molecule inhibitors abrogates generation of human iPSCs

**Figure 2:**
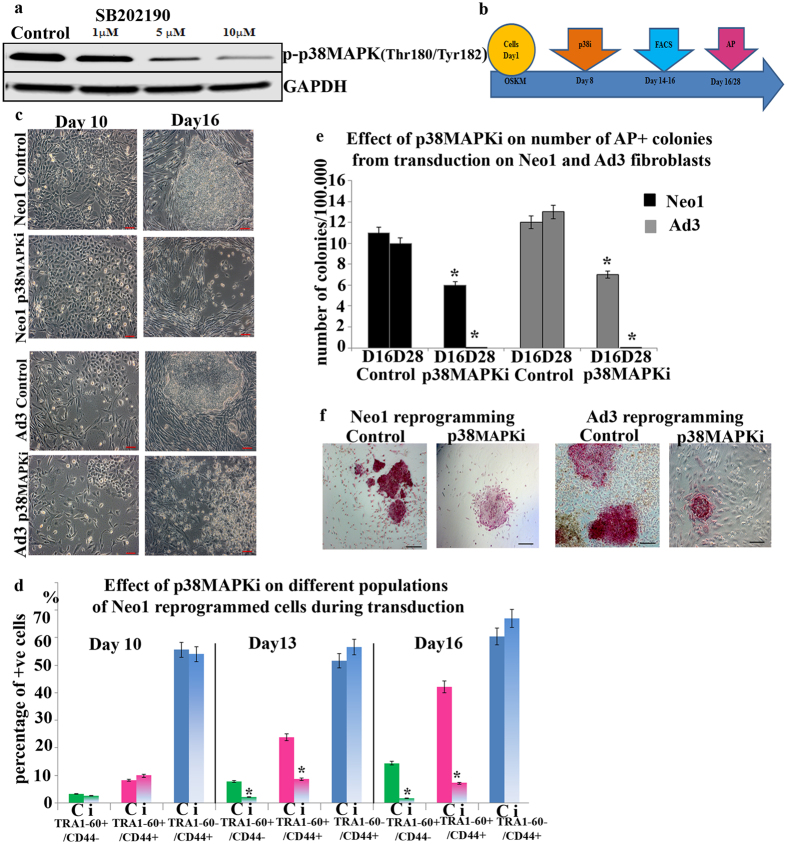
Application of p38MAPK inhibitor (SB202190) abrogates iPSC generation. (**a**) Western blot analysis of p-p38MAPK downregulation by SB202190 (p38MAPKi) in hESCs (H9). GAPDH is used as a loading control. Images are representative of at least three independent experiments. Cropped images of at least three repeats are shown for purpose of clarity; (**b**) Schematic representation of inhibitor application (p38i) at Day 8 during the reprogramming process; **(c)** Phase–contrast observation showing the morphology of partially reprogrammed colonies arising during the reprogramming of Neo1 fibroblasts treated with DMSO or p38MAPKi for 24 hours at Day 8, scale bars = 100 μm; (**d**) Graphic representation of flow cytometric data analysis indicating a significant impact of p38MAPKi (i) application on the percentage of TRA1‐60 + /CD44−, TRA1‐60+/CD44 + and TRA1‐60‐/CD44 + cells at Days 10, 13 and 16. Results are presented as mean ± S.E.M, C: Control; i: p38MAPK inhibitor. Student t-test was carried out to detect significant changes between Control and p38MAPKi group, *p < 0.05; **(e)** Graphic representation of the colony numbers at Days 16 and 28 of reprogramming in p38MAPKi and DMSO treated Neo1 and Ad3 fibroblasts. Data are presented as mean ± S.E.M, n = 3. Student t-test was carried out to detect significant changes between Control and p38MAPKi group, *p < 0.05; (**f**) Alkaline phosphatase staining at Day 16 confirmed the absence of a large AP+ colonies with a typical morphology from Neo1 and Ad3 fibroblasts undergoing reprogramming and treated with p38MAPKi at Day 8 of transduction for 24 hours.

To explore the functions of p38MAPK during somatic cell reprogramming, we utilized pyridinyl imidazole, **SB202190 (p38i),** a small molecule known to inhibit the p38MAPK kinase activity^23^ (Fig. 2a). Given the early activation of the p38 pathway at the early stages of reprogramming in most of the subcellular populations studies (Fig. 1d), we applied this p38 inhibitor at different time points during the first ten days of reprogramming (a day before the start of reprogramming or at days 4 and 8 as shown in Fig. 2b). Regardless of the application timing, we observed the emergence of transduced cells (showing characteristic morphological changes different to those of fibroblasts) which went on to form iPSC colonies in the control group but were detached and lost in the p38 inhibitor treated groups during the maturation stage of reprogramming (Fig. 2b and c). Flow cytometric analysis confirmed a significant reduction in the fraction of partially and fully reprogrammed cells from day 13 in the p38 inhibitor treated cultures of neonatal and adult fibroblasts (Fig. 2d and Suppl. Figure1), and no impact on the fibroblasts themselves (TRA1-60^−^CD44^+^). This was further corroborated by the reduced number of AP + colonies on day 16 and the complete absence of such colonies on day 28 of reprogramming in both neonatal and adult fibroblasts (Fig. 2e and f). Together these data suggest that p38 MAPK activity is required for the development of human iPSC colonies during the maturation stage of reprograming.

### Reprogramming of p38a deficient fibroblasts results in loss of human iPSC colonies

**Figure 3:**
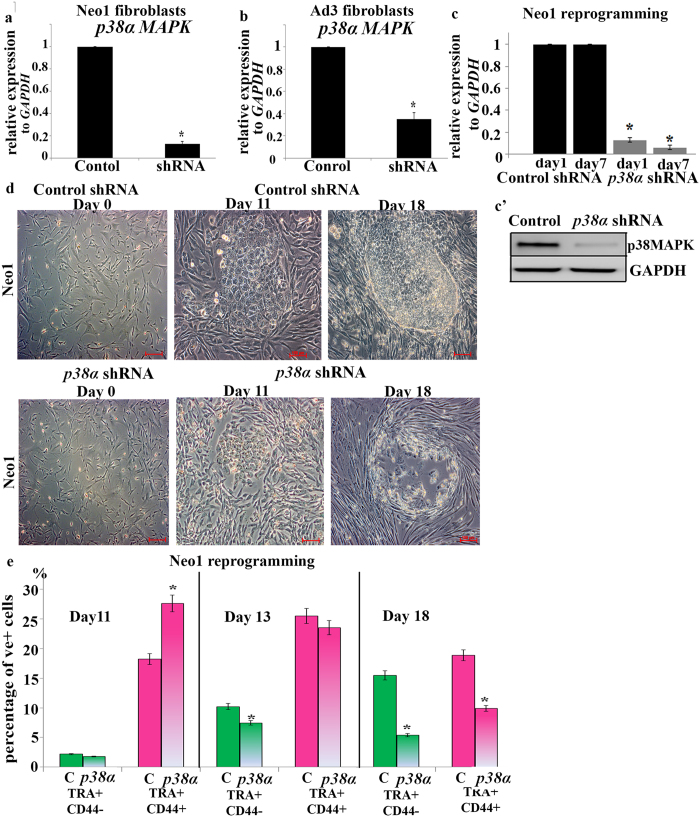
Inhibition of *p38α* MAPK signalling prevents generation of human iPSCs. (**a**) and (**b**) Assessment of *p38α* MAPK downregulation in neonatal (A) and adult (B) fibroblasts by quantitative RT‐PCR analysis. Data represent relative expression to *GAPDH,* normalized against Control shRNA. Results are presented as average ± S.E.M, n = 3, Student t-test was carried out to detect significant changes between Control shRNA and *p38α* shRNA group, *p < 0.05; (**c**) Confirmation of *p38α* MAPK downregulation by quantitative RT‐PCR analysis during reprogramming of Neo1 fibroblast at days 1 and 7. Data represent relative expression to *GAPDH,* normalized against Control shRNA. Results are presented as average ± S.E.M, n = 3, *p < 0.05; (**c’**) Western blot analysis of p38MAPK downregulation by RNAi in *p38α* shRNA transduced Neo1 fibroblasts at day 7. GAPDH is used as a loading control. Images are representative of at least three independent experiments. Cropped images of at least three repeats are shown for purpose of clarity; (**d**) Phase‐contrast images of the Neo1 fibroblasts morphology treated with Control shRNA and *p38α* shRNA at day 0 and colonies arising during reprogramming of the control and *p38α* MAPK deficient Neo1 fibroblasts at Days 11 and 18. Scale bar = 100μm; **(e)** Graphic representation of flow cytometric data analysis indicating a significant impact of *p38α* shRNA downregulation on the percentage of TRA1‐60 + /CD44‐ and TRA1‐60 + /CD44 + cells at Days 13 and 18. Results are presented as mean ± S.E.M, n = 3. Student t-test was carried out to detect significant changes between Control shRNA and *p38α* shRNA group, *p < 0.05.

Application of small molecule inhibitors allows molecular investigations at specific but transient time windows during the reprogramming process. To investigate if continuous reduced levels of p38 MAPK activity affect somatic cell induced reprogramming, we generated stable knockout neonatal and adult fibroblast cell lines by shRNA knockdown of the α isoform of p38MAPK (*p38α* shRNA thereafter) and subjected these to OSKM reprogramming as indicated in the previous section. Gene expression analysis confirmed the successful downregulation of *p38α* in both cell lines (Fig. 3a and b), which was reconfirmed at day 7 of reprogramming both at the level of gene and protein expression (Fig. 3c, c’). By day 11 of reprogramming, iPSC colonies were observed in both the control and *p38α* shRNA transduced fibroblasts; however the latter started to disaggregate by day 13 and were completely lost by day 16 thus corroborating the data obtained with the small molecule inhibitor (Fig. 3d). Flow cytometric analysis demonstrated an initial increase in the percentage of partially reprogrammed cells at day 11. However, the fraction of fully reprogrammed (TRA1-60^+^CD44^−^) cells was significantly reduced at days 13 and 18 of reprogramming when compared to control shRNA cells (Fig. 3d). No AP + colonies were obtained from reprogramming of *p38α* shRNA transduced fibroblasts (data not shown), supporting our findings with the p38 MAPK inhibitor and strongly suggesting that p38 MAPK activity is important for the maturation window of reprogramming.

**Figure 4:**
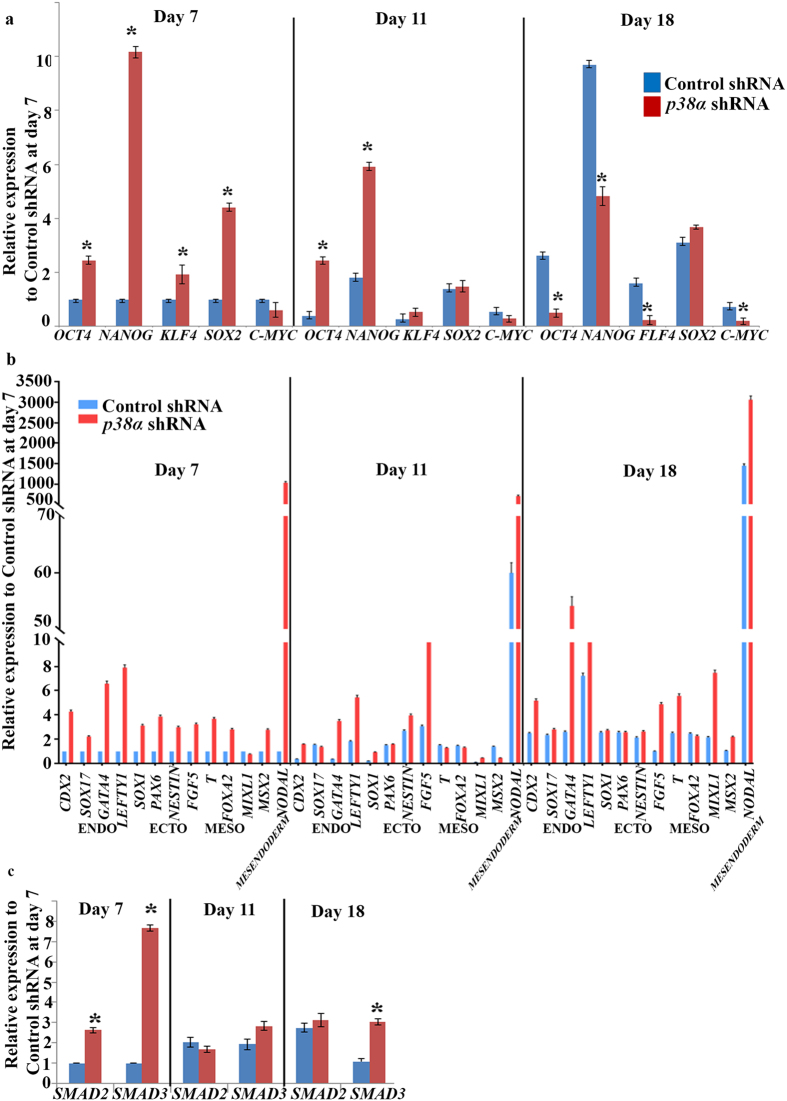
*p38α* MAPK downregulation results in reduced expression of pluripotency genes and precocious expression of mesendodermal markers. (**a**) Quantitative RT-PCR analysis *of OCT4, NANOG, KLF4, SOX2* and *C-MYC* in reprogrammed Neo1 Control shRNA and *p38α* MAPK deficient cells at Days 7, 11 and 18. Results are presented as relative expression and normalised to Control shRNA at day 7. Data are presented as mean ± S.E.M, n = 3. Student t-test was carried out to detect significant changes between Control and *p38α* shRNA group. *p < 0.05; (**b**) Quantitative RT-PCR analysis of *CDX2, SOX17, GATA4, LEFTY1, SOX1, PAX6, NESTIN, FGF5,T, FOXA2, MIXL1, MSX2 and NODAL* during the time course of Neo1 Control shRNA and *p38α* shRNA reprograming. Results are presented as relative expression and normalised to Control shRNA at day 7. Results are presented as mean ± S.E.M, n = 3, *p < 0.05; (**c**) Quantitative RT-PCR analysis of *SMAD2* and *SMAD3* expression at Neo1 Control shRNA and *p38α* shRNA transduced cells at Days 7, 11 and 18. Results are presented as relative expression and normalised to Control shRNA at day 7. Results are presented as mean ± S.E.M, n = 3, *p < 0.05.

To understand how downregulation of p38 MAPK affects reprogramming, we first addressed the expression of pluripotency markers by quantitative RT-PCR analysis (Fig. 4a). The *p38α* shRNA cells expressed pluripotency genes at a higher level on day 7 when compared to control shRNA transduced cells. Some of the pluripotency genes (*OCT4* and *NANOG*) maintained this higher expression at day 11. This trend was however reversed with the majority of the pluripotency genes being significantly downregulated at day 18 which suggests loss of human iPSC colonies from culture, a block in reprogramming process and/or differentiation of newly emerged iPSCs. To investigate this in more detail we performed gene expression analysis with a large number of differentiation markers (**endoderm**: *SOX17, GATA4*; **ectoderm**: *SOX1, PAX6, NESTIN, FGF5;* **mesoderm**: *T, FOXA2, MSX2, MIXL1* and **trophoectoderm**: *CDX2*), the majority of which were significantly upregulated in *p38α* shRNA transduced fibroblasts from day 7 of reprogramming (Fig. 4b). We also paid close attention to two pluripotency and mesendodermal markers, *NODAL* and *LEFTY1*, whose expression has been reported to rise dramatically as cells transit from the initiation to the maturation and stabilization stage of reprogramming^23^. The early upregulation of these two genes during the initiation stage of reprogramming is thought to reflect setting of a mesendodermal gene expression pattern in cell undergoing reprogramming as shown byTakahashi, *et al*.^23^. The second wave of upregulation (day 10 onwards) is thought to reflect the establishment of a pluripotent gene program in emerging and established human iPSC colonies^22^. We were able to observe this pattern of increased *NODAL* and *LEFTY1* expression from day 7 to day 18 of reprogramming in control shRNA cells (Fig. 4b). However, in *p38α* shRNA treated cells, expression of these two markers was very high at the initiation stage of reprogramming (day 7) but did not show the expected drastic upregulation from day 7 to day 18 as shown in control shRNA treated cells (for *LEFTY* 7.2 fold change in control vs 2.7 in the *p38α* shRNA group and for *NODAL* 1454 fold change in control vs 2.96 fold in the *p38α* shRNA group). This suggests that upon p38 MAPK downregulation, the cells undergoing reprogramming do not make the transition from partially reprogrammed cells (characterized by mesendodermal gene expression pattern) to fully reprogrammed cells. It is interesting to note that inhibition of p38 MAPK activity also led to a significant increase in expression of *SMAD2* and *SMAD3*, two key effectors of the TGFβ/Activin A signaling pathway which controls maintenance of pluripotency in hESCs via direct binding and regulation of *NANOG* transcription^24^ as well as priming the expression of tissue specific genes allowing differentiation to mesendodermal lineages^25^. Whilst these data may suggest a direct cross talk between p38 and TGFβ/Activin A signalling as demonstrated by work performed in other cell types^25^,^26^ it may also provide an underlying reason for the enhanced and precocious expression of mesendodermal markers at the initiation stages of reprogramming upon p38 MAPK inhibition as reported herein.

### Downregulation of p38 MAPK activity results in altered cell cycle progression

**Figure 5:**
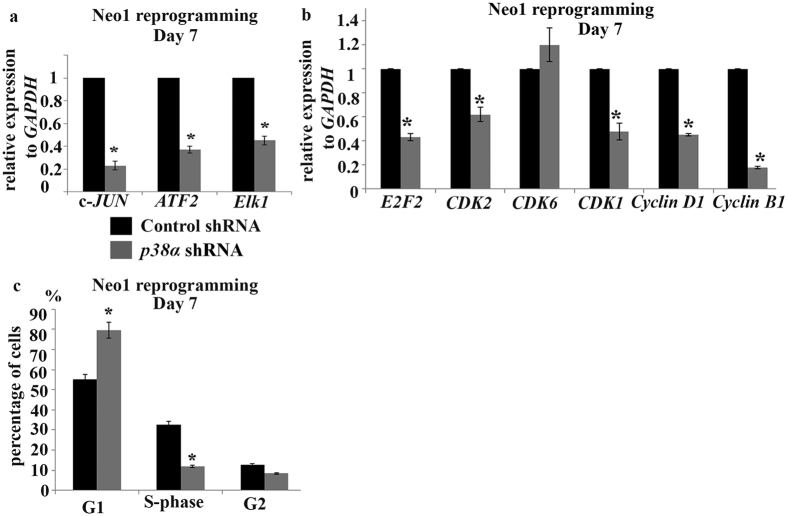
*p38α* MAPK downregulation results in changes in the expression of key genes involved in cell cycle progression and proliferation. (**a**) and (**b**) Quantitative RT-PCR analysis of *c‐JUN, ATF2 and ELK1* (**a**) and *E2F2, CDK2, CDK6, CDK1, CYCLIN D1, CYCLIN B1* (**b**) in Control shRNA and *p38α shRNA* Neo1 transduced cells at Day 7 of reprogramming. Results are presented as mean ± S.E.M, n = 3, *p < 0.05; (**c**) Graphic representation of flow cytometric cell cycle analysis of Control and p38α shRNA treated cells during the reprogramming process at Day 7. Results are presented as mean ± S.E.M, n = 3. *p < 0.05.

Human ESCs proliferate rapidly with a characteristic cell cycle structure of a short G1 phase, which serves to prevent differentiation^27^,^28^ These cells are also characterised by a high CDK activity and downregulation of CDK2 or CDK1 expression induces their irreversible differentiation^28^,^29^ A direct link between maintenance of the pluripotent state and cell cycle regulation is well established. Previously, we demonstrated that Nanog directly regulates *CDK6* and *CDC25A* expression^30^, whilst others described direct regulation of *CDK1* and *CDK2* by OCT4 and SOX2 respectively^31^,^32^. Acceleration of cellular proliferation and acquisition of the typical pluripotent stem cell cycle profile is one of the important prerequisite for successful generation of iPSCs^33^ Hence we examined the expression of the downstream targets of p38MAPK which are important for proliferation and migration of cells (Fig. 5a), namely *c-JUN, ATF2* and *ELK1* which appeared to be downregulated in *p38α* shRNA cells. In addition, the expression of key cyclin dependent kinases and their interacting cyclins were significantly downregulated in *p38α* shRNA group at day 7 of reprogramming (Fig. 5b). Furthermore, flow cytometric cell cycle analysis revealed that about 80% of *p38α* shRNA cells accumulated in G1 phase of the cell cycle (Fig. 5c). Therefore, accumulation in G1 phase of the cell cycle with reduced expression of proliferation markers may be one of the underlying factors for the complete lack of human iPSC colony formation observed upon p38 MAPK downregulation.

### Impairment of MET upon p38 MAPK downregulation

**Figure 6:**
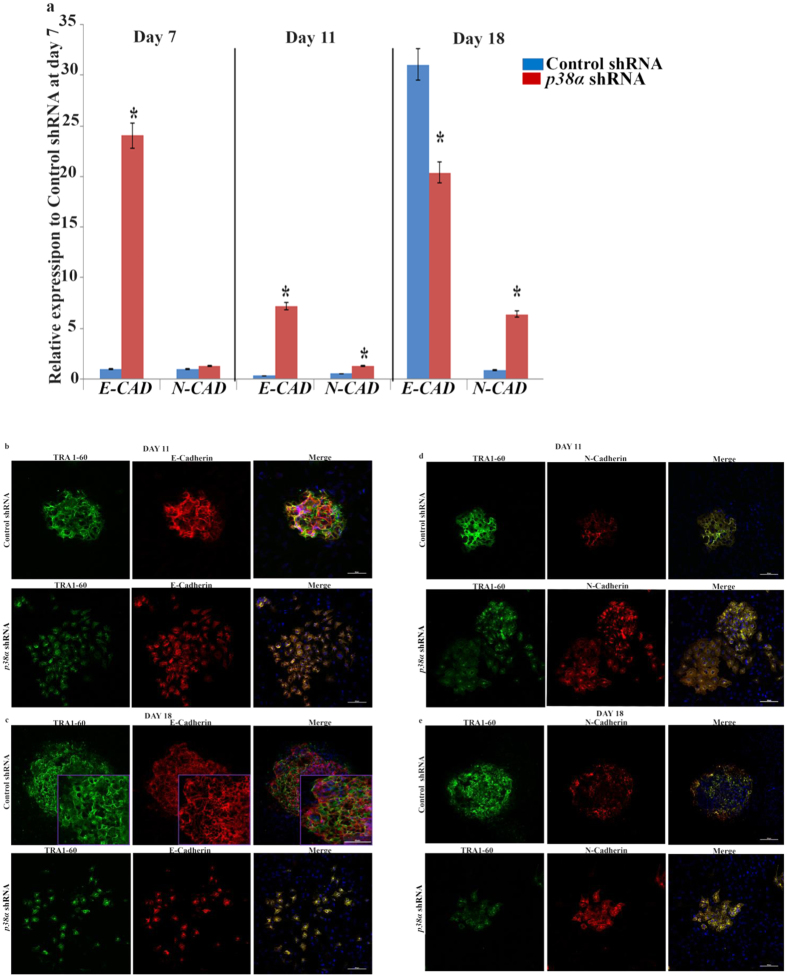
Downregulation *of p38α* MAPK results in alteration of MET progression and increased expression of N‐Cadherin. (**a**) Quantitative RT-PCR analysis of *E-CADHERIN (E-CAD*) and *N-CADHERIN (N-CAD*) expression at Days 7, 11 and 18 in Control and *p38α* shRNA Neo1 reprogrammed fibroblasts. Results are presented as relative expression and normalised to Control shRNA at day 7. Data are presented as mean ± S.E.M, n = 3. Student t-test was carried out to detect significant changes between Control and *p38α* shRNA group. *p < 0.05; (**b–e**) Immunofluorescence staining of transduced Control and *p38α* MAPK deficient Neo1 fibroblast for E‐CADHERIN **(b** and **c),** and N‐CADHERIN (**d** and **e**) together with TRA1‐60 (green) and DAPI (blue) on Days 11 and 18 of reprogramming assessed by confocal microscopy. Scale bars represent 100 μm. Images are representative of at least three independent experiments.

During gastrulation, epithelial to mesenchymal transition (EMT) is necessary for migration of mesodermal cells through the primitive streak in the posterior region of the epiblast. These morphogenetic movements are mediated by regulated changes in cell adhesion^34^ and completion of EMT is dependent on downregulation of E-Cadherin and the acquisition of mesenchymal gene expression. Two pathways act independently to regulate E-Cadherin in the primitive streak to allow migration of mesodermal cells. Fgf2/Snail acts at the transcriptional level to downregulate *E-Cadherin* and p38 MAPK and p38-interacting protein (p38IP) act to downregulate and to destabilize E-Cadherin protein^35^ Thus, p38MAPK and p38IP are required for the downregulation of E-Cadherin during gastrulation. The reprogramming of somatic cells towards iPSCs is critically dependent on the opposite program: mesenchymal to epithelial transition^36^ (MET) which results in the downregulation of mesenchymal markers (N-Cadherin) and upregulation of epithelial markers (E-Cadherin). Based on these previously published data we expected that downregulation of p38 MAPK activity would enhance E-Cadherin expression and promote MET. Indeed, our immunocytochemical and qRT-PCR analysis of genes participating in MET transition revealed a significant upregulation of *E*-*Cadherin* expression at initiation stage of reprogramming (day 7 and day 11) in the *p38α* shRNA group (Fig. 6a); however its expression declined towards day 18 of reprogramming and was also associated with an increase in N-Cadherin expression (Fig. 6a–e), highly suggestive of a disturbed balance between E-cadherin and N-cadherin expression. In contrast, cells treated with control shRNA showed the expected upregulation of *E-Cadherin* expression during the maturation stage of reprogramming indicating progression of MET^37^ It is known that fully reprogrammed iPSCs have high levels of E-Cadherin but lack expression of N-Cadherin, whereas cells that do not completely convert to iPSCs retain N-Cadherin expression^38^ In view of this, increased expression of N-Cadherin could directly reflect the block in the reprogramming process which was highlighted in the previous results section. Maintenance of E-Cadherin expression is also important for cell to cell communication, cell survival and maintenance of pluripotency,^39^,^40^ thereby dysregulation of its expression could explain the loss and disaggregation of iPSC colonies at the maturation stage of reprogramming as well as the onset of differentiation for the remaining adherent cells, which is also supported by decreased expression of pluripotency markers (*KLF4, OCT4, NANOG* and *c-MYC*) at day 18 of reprogramming. In conclusion, our data indicate that MET transition is impaired upon p38 MAPK downregulation leading to loss and disaggregation of “bona fide” iPSC colonies, creating a roadblock in progression of reprogramming and/or encouraging the differentiation of newly formed iPSCs.

## Discussion

The discovery that overexpression of four transcription factors can revert differentiated cells to iPSCs offers immense potential for autologous cell-replacement therapies, drug screening and generation of *in vitro* models of human disease^41^. Despite great advances made in the last ten years, the efficiency of reprogramming remains low and our understanding of the process still incomplete. Large scale shRNA screens have revealed a large number of genes that affect reprogramming as a barriers or enhancers in both human^42^ and mouse cells^43^. Several signalling pathways have been implicated in the reprogramming process, including BMP^44^, G-protein receptor, hypoxia mediated, JAK-STAT, NFkB, NOTCH, TGFβ and FGF2 signalling^7^,^45^,^46^,^47^,^48^ We recently demonstrated an important role for stress activated JNK1/SAPK signalling in overcoming the reprogramming barriers encountered during the initiation stage of reprogramming including acceleration of cellular proliferation and progression of MET^19^. p38α, a key member of stress activated MAPK, is the main isoform expressed in hESC shown to be involved in the maintenance of pluripotency differentiation balance in conjunction with p53 signalling and methionine metabolism^49^. In this manuscript we investigated the role of activated form of p38 MAPK during somatic cell induced reprogramming and found that it is highly expressed in partially reprogrammed human cells in the initiation stage of reprogramming and fully reprogrammed cells in the maturation stages of reprogramming, which implies an important role in transition from the initiation to maturation stage. Indeed, inhibition of this pathway using a small molecule inhibitor applied during the initiation stage resulted in the loss of true bona fide human iPSC colonies and survival of partially reprogrammed cells. Similarly, inhibition of this pathway by RNAi led to cell accumulation in G1 phase of the cell cycle, a significant reduction in expression of proliferation and cell cycle progression markers, maintenance of a higher level of differentiation markers and presence of partially reprogrammed cells which retained a typical gene expression profile of mesendodermal cells and which were unable to progress to fully reprogrammed phenotype as shown by an increased N-cadherin and reduced E-cadherin expression. These results do corroborate some of the work carried out in mouse fibroblasts which show a detrimental role for activators of p38MAPK pathway (namely MKK6) during somatic cell induced reprogramming^6^. At the same time, they contradict recent published data indicating that inhibition of p38β via a small molecule inhibitor increases the efficiency of reprogramming process in MEF^8^. Together, these data suggest potential differences in the role that this pathway may have during the reprogramming of human versus mouse somatic cells which may be related to the different signalling pathways used by mouse and human cells with respect to maintenance and induction of pluripotency^9^,^10^. To be able to elucidate these differences in detail, one will need to perform the experiments side by side in human and mouse cells under identical conditions. Nevertheless, since our research focus is on human pluripotent stem cells and their differentiation to functionally relevant cell types for disease modelling and clinical therapies, in this manuscript we have focused our attention on the role of p38 MAPK during reprogramming of human somatic cells.

**Figure 7:**
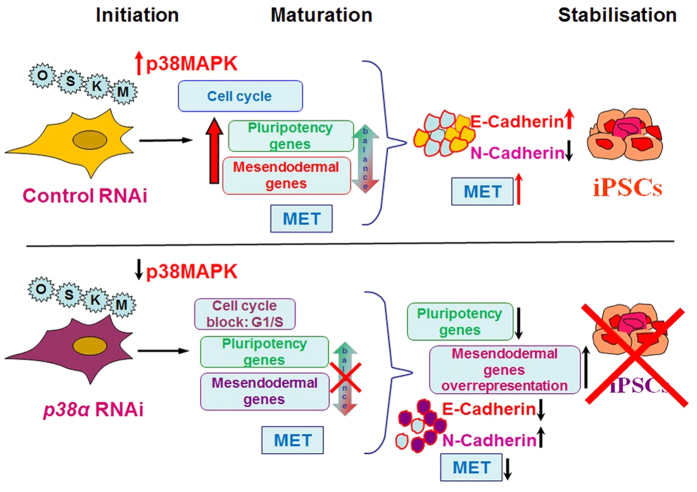
Schematic summary showing the impacts of p38MAPK signalling on iPSC generation.

The increased activity of p38 MAPK observed during the early stages of reprogramming in partially and fully reprogrammed cells as shown in our study may suggest an important role for this pathway in the emergence of partially reprogrammed cells and/or their transition to fully reprogrammed state. Hence we anticipated that downregulation of this pathway would negatively affect generation of iPSC colonies. However, the results we obtained were of both beneficial and harmful nature depending on the time window of reprogramming and cellular subpopulation being investigated. First, the number of partially reprogrammed cells was increased (although they did not form defined colonies with tight borders) upon stable downregulation of p38 MAPK activity. This was associated with an increased expression of pluripotency markers, E-cadherin and SMAD2 and SMAD3, two key effectors of TGFβ/Activin signalling pathway which has been reported to have a significant impact on the maintenance of pluripotency in conjunction with FGF2 signalling via upregulation of NANOG expression. Together these findings suggest that p38 inhibition may have some beneficial impacts at the early stages of reprogramming by activating TGFβ/Activin signalling leading to a higher propensity to generate partially reprogrammed cells as well as enhancing MET transition through the increased E-cadherin expression. Whilst MET is an important factor for successful reprogramming, other key processes including enhanced cellular proliferation and acquisition of a cell cycle profile more akin to iPSC^33^,^50^ are crucially important for the generation of iPSC colonies. Inhibition of p38 MAPK activity had a negative impact on both cellular proliferation and cell cycle progression resulting in cell accumulation in G1 phase of the cell cycle during which cells are more likely to be exposed to differentiation signals. Furthermore, the partially reprogrammed cells were unable to progress to true iPSC or retain the necessary E-cadherin/N-cadherin expression balance which is essential for progression of reprogramming and generation of stable iPSC colonies. These events in combination led to loss of human iPSC colonies and survival of partially reprogrammed cells which retained a higher than normal expression of differentiation markers. Together these data indicate that levels of p38 MAPK activity need to be regulated very precisely during the reprogramming window to ensure maintenance of a fine balance between an enhanced cellular proliferation (which is needed for reprogramming to succeed to the next stage), initiation of MET and setting of a pluripotency gene expression programme, steps which are all critical for the maturation and stabilisation of iPSC colonies as shown in schematic Fig. 7.

It is of interest to note that the expression of two TGFβ/Activin A signalling target genes (NODAL and LEFTY) also increased in the early initiation stage of reprogramming upon downregulation of p38 MAPK activity. In addition to impacting on maintenance of pluripotency, the TGFβ/Activin signalling pathway is as also involved in establishing a mesendodermal gene expression programme. Both of these impacts of TGFβ/Activin signalling are reflected in the increases we have observed in the expression of differentiation markers and in particular the markers that characterise the mesendodermal and pluripotent cells (such as *NODAL* and *LEFTY*). During the reprogramming, cells with a typical profile of mesendodermal cells appear first; however they progress from this partially reprogrammed stage to fully pluripotent phenotype by the stabilisation stage of reprogramming^23^. This transition is marked by gradual increases in expression of *NODAL* and *LEFTY* which we have also observed in our control cultures as they go through the reprogramming process. Notwithstanding this, cells with reduced p38 MAPK activity seem to be “blocked” at this early mesendodermal stage of reprogramming as this gradual increase in of *NODAL* and *LEFTY* expression is not observed during the reprogramming time course. Whilst this phenomenon is disadvantageous for full reprogramming, it can be used to direct conversion of fibroblasts to cell lineages of interest (for example mesendoderm) by adding p38 MAPK inhibitors in the early stages of the conversion process.

Currently it is difficult to reconcile the increased and precocious expression of *SMAD2/3 and NODAL/LEFTY1* in *p38α* deficient cells as it was shown that p38MAPK is required for the maximal Activin/Nodal signalling and for the Smad2 phosphorylation leading to it activity during specification of the anterior visceral endoderm in the mouse post-implantation embryo^25^. A recent report^51^ has indicated that key cell cycle regulators (Cyclin D1-3) control differentiation signals such as the TGFβ/Smad2-3 pathway, hence increased expression of these two effectors could be an indirect consequence of cell accumulation in G1 rather than direct interaction between p38 MAPK and TGFβ signalling pathways. *Smad3* was recently identified as one of the strongest repressors of the hiPSCs generation^51^, thus its increased expression at the maturation stage of reprogramming could be an additional factor for the loss and degradation we observed during reprogramming of p38 deficient fibroblasts. It may be possible that these more extensive impacts we observed upon p38 MAPK inhibition are achieved through changes in DNA methylation as demonstrated during reprogramming of murine fibroblasts undergoing hyperosmosis driven p38 activation^6^ and which merit further investigations for human fibroblast reprogramming. It is also likely that p38 MAPK affect different subcellular populations in different ways and in co-operation with other signalling pathways. For example, cross talk between different signalling pathways can be synergistic (e.g.–p38MAPK and JNK in neural differentiation) or antagonistic (e.g.p38MAPK and ERK in adipocyte differentiation of ESC)^5^. This complexity is further increased by the role of chromatin modifications which are also in part regulated by MAPK during hESC differentiation resulting in lineage commitment decisions as shown by the precocious appearance of partially reprogrammed cells with a mesendodermal gene expression pattern upon p38 inhibition. The data we have obtained in this paper are a clear reflection of complexity one encounters whilst working with heterogeneous and changing cellular subpopulations and highlight the need for further studies in enriched subcellular populations which emerge during the reprogramming process^52^.

In conclusion, we have demonstrated a stage- and subpopulation-specific activation of p38 MAPK activity during somatic cell induced reprogramming of human fibroblasts. Furthermore, we have demonstrated that inhibition of p38 MAPK activity results in cell accumulation in G1 phase, downregulation of important cell cycle regulatory components, disruption of MET, an unbalanced and precocious expression of mesendodermal genes and inability to transit from a partially to fully reprogrammed state leading to loss of human iPSC colonies at the maturation stage of reprogramming as illustrated in Fig. 7, thus demonstrating a critical and indispensable role of this pathway for iPSCs generation.

## Materials and Methods

### hiPSC generation and cell culture

CytoTune‐iPS 2.0 Sendai reprogramming kit (A16517, Invitrogen) was used for IPSC derivation as described recently^19^. Human neonatal and adult dermal fibroblasts were purchased from Lonza and were cultured as described earlier^28^.

#### RNA interference

Stable downregulation of human *p38α* MAPK in human neonatal and adult fibroblasts was carried out using lentiviral based MISSION shRNAs (TRCN0000000510) according to the manufacturer’s protocol (Sigma). Cell culture and selection of the stable clones was performed as described^19^.

#### Western immunoblotting

Protein extraction, western blotting and antibody/antigen complex detection were performed as published previously^28^. Primary antibodies were p38MAPK (Cat N 9212) and phospho-p38MAPK(Thr180/Tyr182) both from Cell Signalling. The antibody to GAPDH (Abcam, ab9485) was used as a loading control.

### Immunocytochemistry and confocal microscopy

Immunocytochemistry was performed as before (Neganova *et al*.^19^). Primary antibodies used in this study were anti-TRA-1-60 FITC conjugate (Merck Millipore), anti-E-Cadherin (Cell Signaling Technology), anti-N-Cadherin (BD Biosciences) and DAPI (diamidino-2-phenylindole) was from Thermo Fisher. The images were acquired with a Nikon A1R laser scanning confocal microscope (Nikon, http://nikon.com) using a CFl Plan Apochromat VC 20×/0.75 objective as described^19^.

#### Quantitative RT-PCR

Cells were harvested and total RNA was extracted using TRIzol (Invitrogen, 15596–026), according to manufacturer’s instructions. All steps performed as described before^19^. Samples were normalised using *GAPDH*. All DNA oligonucleotide sequences are listed in Suppl. Table 1.

#### Flow cytometric analysis

Cells were disassociated using Versene (EDTA) (Lonza), washed with PBS and fixed in paraformaldehyde (PFA; 2% final concentration in PBS) at 37 °C for 10 minutes. After washing with PBS, the cells were permeabilised with pre-chilled methanol (−18 °C) and incubated at 4 °C for 30 minutes, followed by a washing step. 0.2–0.5 × 10^6^ cells were resuspended in a total volume of 200 μl PBS containing 1% BSA and incubated with appropriate amounts of anti-CD44-BV421 (Cat.N.562890, BD Biosciences; 1:300 dilution), anti-TRA-1-60-FITC (Cat. N. FCMAB115F, Merck Millipore; 1:100 dilution) and Phospho-p38 MAPK (Cat. N. 4552, Cell Signaling Technology; 1:50 dilution; Mouse mAb Ig1 Isotype Control Alexa Fluor 647 Conjugate Cat. N.4843, Cell Signaling) monoclonal antibodies for 1 hour on a shaker, plate in the dark at room temperature. Finally, samples were washed using BD FACS™ Lyse Wash Assistant (BD Biosciences) and immediately analysed on a flow cytometer. FACS analysis was performed using BD FACS Canto II flow cytometer with FACSDiva software (BD Biosciences). A minimum of 20,000 events were recorded for each sample. Fluorescence minus One control (for each antibody) was used to gate the subpopulations.

**A**lkaline Phosphatase detection was performed with Alkaline Phosphotase detection kit (SCR004, Millipore) according to manufacture instructions.

#### Statistical analysis

Student t‐test analysis was used to assess differences between Control and *p38α* shRNA groups. The results were considered significant if *p* < 0.05.

## Additional Information

**How to cite this article**: Neganova, I. *et al*. A critical role for p38MAPK signalling pathway during reprogramming of human fibroblasts to iPSCs. *Sci. Rep.* **7**, 41693; doi: 10.1038/srep41693 (2017).

**Publisher's note:** Springer Nature remains neutral with regard to jurisdictional claims in published maps and institutional affiliations.

## Supplementary Material

Supplementary Information

## References

[b1] Cuadrado A. & Nebreda A. R. Mechanisms and functions of p38 MAPK signalling. The Biochemical journal 429, 403–417, doi: 10.1042/BJ20100323 (2010). 20626350

[b2] Allen M. . Deficiency of the stress kinase p38alpha results in embryonic lethality: characterization of the kinase dependence of stress responses of enzyme-deficient embryonic stem cells. The Journal of experimental medicine 191, 859–870 (2000). 10704466 10.1084/jem.191.5.859 PMC2195860

[b3] Adams R. H. . Essential role of p38alpha MAP kinase in placental but not embryonic cardiovascular development. Molecular cell 6, 109–116 (2000). 10949032

[b4] Chakraborty S., Kang B., Huang F. & Guo Y. L. Mouse embryonic stem cells lacking p38alpha and p38delta can differentiate to endothelial cells, smooth muscle cells, and epithelial cells. Differentiation; research in biological diversity 78, 143–150, doi: 10.1016/j.diff.2009.05.006 (2009). 19539422 PMC2761660

[b5] Aouadi M. . p38 mitogen-activated protein kinase activity commits embryonic stem cells to either neurogenesis or cardiomyogenesis. Stem Cells 24, 1399–1406, doi: 10.1634/stemcells.2005-0398 (2006). 16424397

[b6] Xu X. . Stress-mediated p38 activation promotes somatic cell reprogramming. Cell research 23, 131–141, doi: 10.1038/cr.2012.143 (2013). 23044805 PMC3541653

[b7] Fritz A. L., Mao S. R., West M. G. & Schaffer D. V. A medium-throughput analysis of signaling pathways involved in early stages of stem cell reprogramming. Biotechnology and bioengineering 112, 209–219, doi: 10.1002/bit.25336 (2015). 25065366

[b8] Li Z. & Rana T. M. A kinase inhibitor screen identifies small-molecule enhancers of reprogramming and iPS cell generation. Nature communications 3, 1085, doi: 10.1038/ncomms2059 (2012). PMC3658009 23011139

[b9] Armstrong L. . The role of PI3K/AKT, MAPK/ERK and NFkappabeta signalling in the maintenance of human embryonic stem cell pluripotency and viability highlighted by transcriptional profiling and functional analysis. Human molecular genetics 15, 1894–1913, doi: 10.1093/hmg/ddl112 (2006). 16644866

[b10] Li J. . MEK/ERK signaling contributes to the maintenance of human embryonic stem cell self-renewal. Differentiation; research in biological diversity 75, 299–307, doi: 10.1111/j.1432-0436.2006.00143.x (2007). 17286604

[b11] Qi X. . BMP4 supports self-renewal of embryonic stem cells by inhibiting mitogen-activated protein kinase pathways. Proceedings of the National Academy of Sciences of the United States of America 101, 6027–6032, doi: 10.1073/pnas.0401367101 (2004). 15075392 PMC395917

[b12] Bulavin D. V. & Fornace A. J. Jr. p38 MAP kinase’s emerging role as a tumor suppressor. Advances in cancer research 92, 95–118, doi: 10.1016/S0065-230X(04)92005-2 (2004). 15530558

[b13] Bulavin D. V., Kovalsky O., Hollander M. C. & Fornace A. J. Jr Loss of oncogenic H-ras-induced cell cycle arrest and p38 mitogen-activated protein kinase activation by disruption of Gadd45a. Molecular and cellular biology 23, 3859–3871 (2003). 12748288 10.1128/MCB.23.11.3859-3871.2003 PMC155214

[b14] Wang W. . Sequential activation of the MEK-extracellular signal-regulated kinase and MKK3/6-p38 mitogen-activated protein kinase pathways mediates oncogenic ras-induced premature senescence. Molecular and cellular biology 22, 3389–3403 (2002). 11971971 10.1128/MCB.22.10.3389-3403.2002 PMC133789

[b15] Han J. & Sun P. The pathways to tumor suppression via route p38. Trends in biochemical sciences 32, 364–371, doi: 10.1016/j.tibs.2007.06.007 (2007). 17624785

[b16] Serrano M., Lin A. W., McCurrach M. E., Beach D. & Lowe S. W. Oncogenic ras provokes premature cell senescence associated with accumulation of p53 and p16INK4a. Cell 88, 593–602 (1997). 9054499 10.1016/s0092-8674(00)81902-9

[b17] Banito A. & Gil J. Induced pluripotent stem cells and senescence: learning the biology to improve the technology. EMBO reports 11, 353–359, doi: 10.1038/embor.2010.47 (2010). 20379220 PMC2868548

[b18] Kumar S. M. . Acquired cancer stem cell phenotypes through Oct4-mediated dedifferentiation. Oncogene 31, 4898–4911, doi: 10.1038/onc.2011.656 (2012). 22286766 PMC3343184

[b19] Neganova I. . JNK/SAPK signaling is essential for efficient reprogramming of human fibroblasts to induced pluripotent stem cells. Stem Cells 34, 1198–1212, doi: 10.1002/stem.2327 (2016). 26867034 PMC4982072

[b20] Chia N. Y. . A genome-wide RNAi screen reveals determinants of human embryonic stem cell identity. Nature 468, 316–320, doi: 10.1038/nature09531 (2010). 20953172

[b21] Kyriakis J. M. & Avruch J. Mammalian MAPK signal transduction pathways activated by stress and inflammation: a 10-year update. Physiological reviews 92, 689–737, doi: 10.1152/physrev.00028.2011 (2012). 22535895

[b22] David L. & Polo J. M. Phases of reprogramming. Stem cell research 12, 754–761, doi: 10.1016/j.scr.2014.03.007 (2014). 24735951

[b23] Takahashi K. . Induction of pluripotency in human somatic cells via a transient state resembling primitive streak-like mesendoderm. Nature communications 5, 3678, doi: 10.1038/ncomms4678 (2014). 24759836

[b24] Vallier L. . Activin/Nodal signalling maintains pluripotency by controlling Nanog expression. Development 136, 1339–1349, doi: 10.1242/dev.033951 (2009). 19279133 PMC2687465

[b25] Clements M., Pernaute B., Vella F. & Rodriguez T. A. Crosstalk between Nodal/activin and MAPK p38 signaling is essential for anterior-posterior axis specification. Current biology: CB 21, 1289–1295, doi: 10.1016/j.cub.2011.06.048 (2011). 21802298 PMC3209556

[b26] Lee K. L. . Graded Nodal/Activin signaling titrates conversion of quantitative phospho-Smad2 levels into qualitative embryonic stem cell fate decisions. PLoS genetics 7, e1002130, doi: 10.1371/journal.pgen.1002130 (2011). 21731500 PMC3121749

[b27] Becker K. A. . Self-renewal of human embryonic stem cells is supported by a shortened G1 cell cycle phase. Journal of cellular physiology 209, 883–893, doi: 10.1002/jcp.20776 (2006). 16972248

[b28] Neganova I., Zhang X., Atkinson S. & Lako M. Expression and functional analysis of G1 to S regulatory components reveals an important role for CDK2 in cell cycle regulation in human embryonic stem cells. Oncogene 28, 20–30, doi: 10.1038/onc.2008.358 (2009). 18806832

[b29] Neganova I. . CDK1 plays an important role in the maintenance of pluripotency and genomic stability in human pluripotent stem cells. Cell death & disease 5, e1508, doi: 10.1038/cddis.2014.464 (2014). 25375373 PMC4260724

[b30] Zhang X. . A role for NANOG in G1 to S transition in human embryonic stem cells through direct binding of CDK6 and CDC25A. The Journal of cell biology 184, 67–82, doi: 10.1083/jcb.200801009 (2009). 19139263 PMC2615089

[b31] Li W. . Identification of Oct4-activating compounds that enhance reprogramming efficiency. Proceedings of the National Academy of Sciences of the United States of America 109, 20853–20858, doi: 10.1073/pnas.1219181110 (2012). 23213213 PMC3529047

[b32] Ouyang J. . Cyclin-dependent kinase-mediated Sox2 phosphorylation enhances the ability of Sox2 to establish the pluripotent state. The Journal of biological chemistry 290, 22782–22794, doi: 10.1074/jbc.M115.658195 (2015). 26139602 PMC4566249

[b33] Ruiz S. . A high proliferation rate is required for cell reprogramming and maintenance of human embryonic stem cell identity. Current biology: CB 21, 45–52, doi: 10.1016/j.cub.2010.11.049 (2011). 21167714 PMC3034649

[b34] Shook D. & Keller R. Mechanisms, mechanics and function of epithelial-mesenchymal transitions in early development. Mechanisms of development 120, 1351–1383 (2003). 14623443 10.1016/j.mod.2003.06.005

[b35] Zohn I. E. . p38 and a p38-interacting protein are critical for downregulation of E-cadherin during mouse gastrulation. Cell 125, 957–969, doi: 10.1016/j.cell.2006.03.048 (2006). 16751104

[b36] Hoffding M. K. & Hyttel P. Ultrastructural visualization of the Mesenchymal-to-Epithelial Transition during reprogramming of human fibroblasts to induced pluripotent stem cells. Stem cell research 14, 39–53, doi: 10.1016/j.scr.2014.11.003 (2015). 25506910

[b37] Li D. . Integrated biochemical and mechanical signals regulate multifaceted human embryonic stem cell functions. The Journal of cell biology 191, 631–644, doi: 10.1083/jcb.201006094 (2010). 20974810 PMC3003326

[b38] Pieters T. & van Roy F. Role of cell-cell adhesion complexes in embryonic stem cell biology. Journal of cell science 127, 2603–2613, doi: 10.1242/jcs.146720 (2014). 24931943

[b39] Redmer T. . E-cadherin is crucial for embryonic stem cell pluripotency and can replace OCT4 during somatic cell reprogramming. EMBO reports 12, 720–726, doi: 10.1038/embor.2011.88 (2011). 21617704 PMC3128971

[b40] Li L., Bennett S. A. & Wang L. Role of E-cadherin and other cell adhesion molecules in survival and differentiation of human pluripotent stem cells. Cell adhesion & migration 6, 59–70, doi: 10.4161/cam.19583 (2012). 22647941 PMC3364139

[b41] Takahashi K. & Yamanaka S. Induction of pluripotent stem cells from mouse embryonic and adult fibroblast cultures by defined factors. Cell 126, 663–676, doi: 10.1016/j.cell.2006.07.024 (2006). 16904174

[b42] Qin H. . Systematic identification of barriers to human iPSC generation. Cell 158, 449–461, doi: 10.1016/j.cell.2014.05.040 (2014). 25036638 PMC4130998

[b43] Yang C. S., Chang K. Y. & Rana T. M. Genome-wide functional analysis reveals factors needed at the transition steps of induced reprogramming. Cell reports 8, 327–337, doi: 10.1016/j.celrep.2014.07.002 (2014). 25043178 PMC4152236

[b44] Samavarchi-Tehrani P. . Functional genomics reveals a BMP-driven mesenchymal-to-epithelial transition in the initiation of somatic cell reprogramming. Cell stem cell 7, 64–77, doi: 10.1016/j.stem.2010.04.015 (2010). 20621051

[b45] Tang Y. . Jak/Stat3 signaling promotes somatic cell reprogramming by epigenetic regulation. Stem Cells 30, 2645–2656, doi: 10.1002/stem.1225 (2012). 22968989

[b46] Soria-Valles C. . NF-kappaB activation impairs somatic cell reprogramming in ageing. Nature cell biology 17, 1004–1013, doi: 10.1038/ncb3207 (2015). 26214134

[b47] Ichida J. K. . Notch inhibition allows oncogene-independent generation of iPS cells. Nature chemical biology 10, 632–639, doi: 10.1038/nchembio.1552 (2014). 24952596 PMC4310751

[b48] Jiao J. . Promoting reprogramming by FGF2 reveals that the extracellular matrix is a barrier for reprogramming fibroblasts to pluripotency. Stem Cells 31, 729–740, doi: 10.1002/stem.1318 (2013). 23307593

[b49] Shiraki N. . Methionine metabolism regulates maintenance and differentiation of human pluripotent stem cells. Cell metabolism 19, 780–794, doi: 10.1016/j.cmet.2014.03.017 (2014). 24746804

[b50] Boward B., Wu T. M. & Dalton S. Concise Review: Control of Cell Fate Through Cell Cycle and Pluripotency Networks. Stem Cells 34, 1427–1436, doi: 10.1002/stem.2345 (2016). 26889666 PMC5201256

[b51] Toh C. X. D. . RNAi Reveals Phase-Specific Global Regulators of Human Somatic Cell Reprogramming. Cell Rep 15, 2597–2607, doi: 10.1016/j.celrep.2016.05.049 (2016). 27292646

[b52] Tonge P. D. . Divergent reprogramming routes lead to alternative stem-cell states (vol 516, pg 192, 2014). Nature 523, doi: 10.1038/nature14607 (2015). 25503232

