# Supplementary material for: A critical role for p38MAPK signalling pathway during reprogramming of human fibroblasts to iPSCs
Source: Sci Rep. 2017 Feb 3;7:41693. doi: 10.1038/srep41693 (PMC5290526; doi:10.1038/srep41693)
Supplement: Supplementary Information [file srep41693-s1.pdf]

Supplement Information for the paper:

A critical role for p38MAPK signalling pathway during reprogramming of human fibroblasts to iPSCs

Irina Neganova[1], Valeria Chichagova[1], Lyle Armstrong[1] and Majlinda Lako [1#]

Supplement Figure 1.

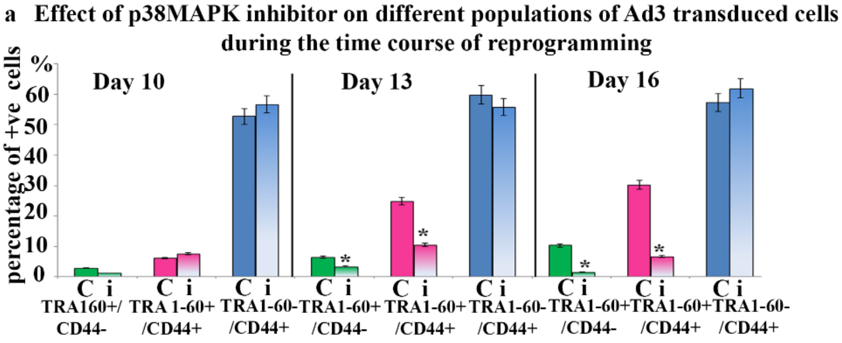

Supplement Figure 1. Application of p38MAPK inhibitor (SB202190) abrogates iPSC generation from adult fibroblasts (a) Graphic representation of flow cytometric analysis data indicating a significant impact of *p38α* MAPK downregulation on the percentage of TRA1-60+/CD44- and TRA1-60+/CD44+ cells at Days 13 and 18. Results are presented as mean ±S.E.M, n=3. Student t-test was carried out to detect significant changes between Control and p38MAPK inhibitor (p38MAPKi) group. * p <0.05.

Supplement Figure 2.

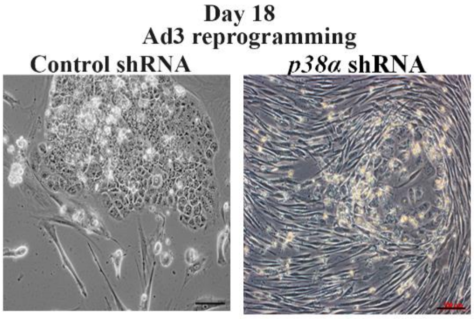

Supplement Figure 2. Inhibition of *p38α* MAPK signalling prevents generation of iPSCs from adult fibroblasts. (a) Phase-contrast images of the colony morphology developed at Day 18 from the Ad3 Control shRNA and *p38α* shRNA transduced cells. Scale bar =100µm.

Supplement Table 1

Gene	Forward primer sequence (5'→3')	Reverse primer sequence (5'→3')
MKK3	CGGCTGCAAGCCCTACAT	CTCCAGACGTCGGACTTGACA
MKK6	GGCCCCTGAAAGAATAAACCC	CGAAGGATGGCCAACTCAATC
p38α	GGGTTACGTGTGGCAGTGAA	ACGTCCAACAGACCAATCACA
OCT4	GAGAACCGAGTGAGAGGCAACC	CATAGTCGCTGCTTGATCGCTTG
NANOG	AATACCTCAGCCTCCAGCAGATG	TGCGTCACACCATTGCTATTCTTC
KLF4	TTACCAAGAGCTCATGCCACC	GCGAATTTCCATCCACAGCC
SOX2	TTGTTCGATCCCAACTTTCC	ACATGGATTCTCGGCAGACT
cMYC	CACCAGCAGCGACTCTGA	CTGTGAGGAGGTTTGCTGTG
CDX2	CTCGGCAGCCAAGTGAAAAC	CTCCTTTGCTCTGCGGTTCT
SOX17	CGCACGGAATTTGAACAGTA	GGATCAGGGACCTGTCACAC
GATA4	TCCAAACCAGAAAACGGAAG	AAGGCTCTCACTGCCTGAAG
LEFTY1	AGAGCTGGCGATGACTGAAC	AAACTGAGCAAGGGCTCTCC
SOX1	GGAATGGGAGGACAGGATTT	ACTTTTATTTCTCGGCCCGT
PAX6	GCCTATGCAACCCCCAGT	TCACTTCCGGGAACTTGAAC
NESTIN	GAGAGGGAGGACAAAGTCCC	CCACTTCCTCAGACTGCTCC
FGF5	CACTGATAGGAACCCTAGAGGC	CAGATGGAAACCGATGCCC
T	CAGTGGCAGTCTCAGGTTAAGAAGGA	CGCTACTGCAGGTGTGAGCAA
FOXA2	GCATTCCCAATCTTGACACGGTGA	GCCCTTGCAGCCAGAATACACATT
MIXL1	GAGACTTGGCACGCCTGT	GGTACCCCGACATCCACTT
MSX2	TGGATGCAGGAACCCGG	AGGGCTCATATGTCTTGGCG
NODAL	GTACATGCTGAGCCTCTACCG	CAAAAGCAAACGTCCAGTTCTG
SMAD3	TCCAGTCTCCCAACTGTAACC	TGTCTCCTGTACTCCGCTC
SMAD2	CCCTCACTCACTGTAGATGG	CACTCCTCTTCCTATATGCCT
c-JUN	GCTCTGGGAAGTGAGTTCG	CTCCCGCACTCTTACTTGTC
ATF2	GCCAATTGT//CCCTGTACCA	GTCCTAACCAATCCGCTACC
ELK1	TCCTACGCATACATTGACCC	ACTGGATGGAAACTGGAAGG
E2F2	AGGAGCTGATGAACACGGA	CGGCAATCACTGTCTGCT
CDK2	TGTACCTCCCCTGGATGAAG	CATCCTGGAAGAAAGGGTGA
CDK6	AGGATAAGCCAACCTGAGAC	ACTGAGAGTATGACTGGCAA
CDK1	TTTTCAGAGCTTTGGGCACT	CCATTTTGCCAGAAATTCGT
CCND1	GGCGGAGGAGAACAAACAG	AGGCGGTAGTAGGACAGGA
CCNB1	AACTTTCGCCTGAGCCTATTTT	TTGGTCTGACTGCTTGCTCTT
E-CADHERIN	TGCCCAGAAAATGAAAAAGG	GTGTATGTGGCAATGCGTTC
N-CADHERIN	ACAGTGGCCACCTACAAAGG	CCGAGATGGGGTTGATAATG
GAPDH	TGCACCACCAACTGCTTAGC	GGCATGGACTGTGGTCATGAG
